# Prophylactic Therapy with Human Amniotic Fluid Stem Cells Improves Long-Term Cognitive Impairment in Rat Neonatal Sepsis Survivors

**DOI:** 10.3390/ijms21249590

**Published:** 2020-12-16

**Authors:** Yushi Abe, Daigo Ochiai, Yu Sato, Seiji Kanzaki, Satoru Ikenoue, Yoshifumi Kasuga, Mamoru Tanaka

**Affiliations:** 1 Department of Obstetrics & Gynecology, Keio University School of Medicine, Tokyo 160-8582, Japan; y.abe@keio.jp (Y.A.); yu.1221.you@gmail.com (Y.S.); skanzaki@stemcell.co.jp (S.K.); ikenouesatoru@me.com (S.I.); 17yoshi23.k@gmail.com (Y.K.); mtanaka@keio.jp (M.T.); 2 StemCell Institute Inc., Tokyo 105-0004, Japan

**Keywords:** amniotic fluid stem cells, neonatal sepsis, prophylactic therapy, hippocampus

## Abstract

A systemic inflammatory response induces multiple organ dysfunction and results in poor long-term neurological outcomes in neonatal sepsis. However, there is no effective therapy for treating or preventing neonatal sepsis besides antibiotics and supportive care. Therefore, a novel strategy to improve neonatal sepsis-related morbidity and mortality is desirable. Recently, we reported that prophylactic therapy with human amniotic stem cells (hAFSCs) improved survival in a rat model of lipopolysaccharide (LPS)-induced neonatal sepsis through immunomodulation. Besides improving the mortality, increasing survival without major morbidities is an important goal of neonatal intensive care for neonatal sepsis. This study investigated long-term neurological outcomes in neonatal sepsis survivors treated with hAFSCs using the LPS-induced neonatal sepsis model in rats. We found that prophylactic therapy with hAFSCs improved spatial awareness and memory-based behavior in neonatal sepsis survivors at adolescence in rats. The treatment suppressed acute reactive gliosis and subsequently reduced astrogliosis in the hippocampal region over a long period of assessment. To the best of our knowledge, this is the first report that proves the concept that hAFSC treatment improves cognitive impairment in neonatal sepsis survivors. We demonstrate the efficacy of hAFSC therapy in improving the mortality and morbidity associated with neonatal sepsis.

## 1. Introduction

Recent advances in neonatal care have significantly improved the survival rate of preterm infants, with low birth weight or with medical conditions. However, the mortality rate of neonatal sepsis is very high, being 10–30% [1,2,3]. Moreover, neonatal sepsis survivors often suffer from severe functional disabilities for a long time. The incidence of neonatal sepsis is inversely correlated with birth weight, and it has been reported that 4–14% of the neurological sequelae among low-birth-weight infants are caused by neonatal sepsis [4]. Sepsis is evoked by an imbalance in host immune response against invasive microorganisms. A systemic inflammatory response induces multiple organ dysfunction and can often cause hypoperfusion, hypoxia, and free radical damage to the brain, resulting in poor long-term neurological outcomes [5,6,7,8]. To date, a number of treatments for neonatal sepsis have been evaluated. However, there is presently no effective therapy for the treatment or prevention of neonatal sepsis besides antibiotics and supportive care. Therefore, it is important to develop innovative and efficacious strategies to reduce neonatal sepsis-related morbidity and mortality.

Mesenchymal stem cells (MSCs) provide a promising platform for cell-based therapy. Owing to their immunomodulatory properties, MSCs are being investigated for prevention and treatment of immune-related diseases, including sepsis. Human amniotic fluid stem cells (hAFSCs) are MSCs derived from the fetus, which can be established before birth [9,10]. They have high proliferative potential and anti-inflammatory and immunomodulatory properties [11,12,13]. Recently, we reported that prophylactic therapy with hAFSCs improved survival in a rat model of lipopolysaccharide (LPS)-induced neonatal sepsis through immunomodulation. The treatment was effective in reducing systemic inflammation and in improving mortality in neonatal rats 48 h after LPS exposure [14]. Besides improving mortality, increasing survival without major morbidities in neonatal sepsis is another important goal of neonatal intensive care. There are concerns that improved survival of infants treated with hAFSCs might be accompanied by an increase in disabling morbidity in survivors. However, the long-term outcomes in neonatal sepsis survivors treated with hAFSCs remain to be elucidated.

The aim of this study was to determine the long-term neurological outcomes, especially spatial awareness and memory-related behavioral outcomes, in neonatal sepsis survivors after hAFSC treatment using a rat model of LPS-induced neonatal sepsis.

## 2. Results

### 2.1. Phenotypic Characterization of hAFSCs

The hAFSCs used in this study were evaluated for differentiation potential and surface markers to ensure that they met the definition of MSCs. As described in our previous report [14], hAFSCs did not express hematopoietic surface markers (CD34, CD14, and CD45) but expressed mesenchymal markers (CD90, CD73, and CD105). They also exhibited the ability to differentiate into osteocytes, chondrocytes, and adipocytes.

### 2.2. hAFSC Administration Reduced the Glial Fibrillary Acidic Protein (GFAP)-Positive Area after LPS Administration

LPS administration induced neuroinflammation in the rat brain, as indicated by GFAP- and ionized calcium-binding adapter molecule 1 (Iba-1)-positive cells. As reported previously [14], LPS injection elicited neuroinflammation 48 h after LPS exposure, whereas the inflammatory changes were significantly attenuated by hAFSC treatment. Although we previously reported a reduction in neuroinflammation in the entire hippocampus after hAFSC treatment, in this study, we analyzed neuroinflammation in three hippocampal regions, viz., CA1, CA3, and dentate gyrus (DG).

**Figure 1:**
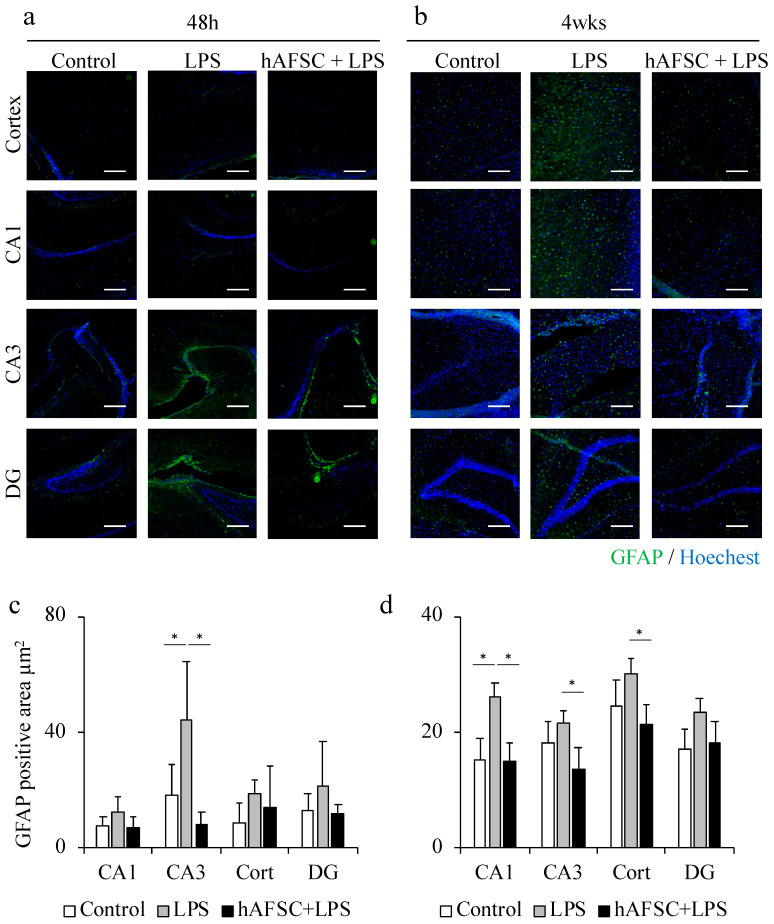
Representative images of immunofluorescence staining for glial fibrillary acidic protein (GFAP) in the hippocampus (CA1, CA3, dentate gyrus (DG) regions) and cerebral cortex of rats at 48 h (**a**) and 4 weeks (**b**) after lipopolysaccharide (LPS) administration. A graph showing the mean GFAP-positive area (**c**) and (**d**), with a significant difference in the CA3 region at 48 h (**c**). This difference was still observed after 4 weeks (**d**). Data are means ± SEM (*n* = 5). * *p* < 0.05. Scale bar = 100 μm.

In the CA3 region, the LPS group had significantly larger GFAP-positive area than the control group, and the hAFSC treatment significantly attenuated the LPS-induced overexpression of GFAP. A similar trend was observed in the CA1 and DG regions, but there was no significant difference between the groups (Figure 1a,c). The same experiment was conducted in individuals that survived for more than 48 h after LPS administration and were maintained for 4 weeks. The GFAP-positive area was significantly greater in the CA1 region in the LPS group than in the control group; however, this effect was significantly inhibited by hAFSC treatment (Figure 1b,d).

### 2.3. hAFSC Administration Reduced Iba-1-Positive Area after LPS Administration

**Figure 2:**
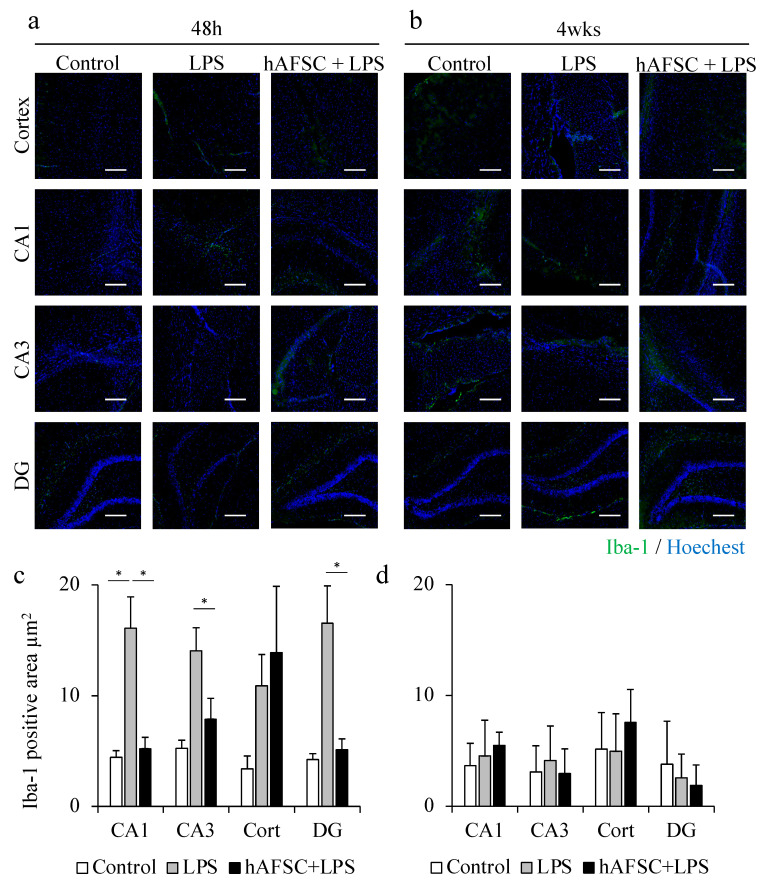
Representative images of immunofluorescence staining for ionized calcium-binding adapter molecule 1 (Iba-1) in the hippocampus (CA1, CA3, dentate gyrus (DG) regions) and cerebral cortex of rats at 48 h (**a**) and 4 weeks (**b**) after lipopolysaccharide (LPS) administration. A graph showing the mean Iba-1-positive area (**c**) and (**d**), with a significant difference in the CA1, CA3, and DG regions at 48 h (**c**). However, after 4 weeks, there was no difference between any of the groups (**d**). Data are means ± SEM (*n* = 5). * *p* < 0.05. Scale bar = 100 μm.

We previously reported that LPS treatment increased the number of Iba-1-positive cells in the rat brain, 48 h after LPS treatment [14]. In the present study, as described above, we also determined the Iba-1-positive area in the hippocampal CA1, CA3, and DG regions. At 48 h after LPS challenge, sepsis increased the Iba-1-positive area in the CA1, CA3, and DG regions, compared with that in the control group. However, hAFSC administration prevented microglial activation in these hippocampal regions (Figure 2a,c).

However, the overexpression of Iba-1 elicited by the LPS challenge was not observed 4 weeks after the exposure. There was no significant difference between the three groups in the Iba-1-positive area in all the regions, 4 weeks after sepsis. These results suggest that microglial activation was suppressed at an earlier time during the process of healing of sepsis, whereas the activation of astrocytes, which is represented by GFAP-positive cells, lasted for a long time (Figure 2b,d).

### 2.4. hAFSC Administration Did Not Affect Dysmyelination

**Figure 3:**
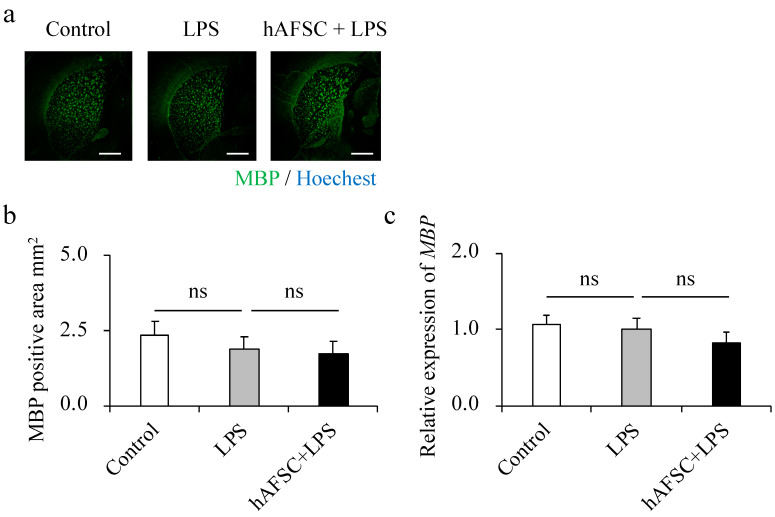
Representative images of immunofluorescence staining for MBP in the white matter at 42 days of age (**a**). There were no significant differences between any of the groups in the MBP-positive area (**b**) and in the expression of MBP (**c**). Data are means ± SEM (*n* = 5). Scale bar = 1 mm.

In previous reports, degradation of white matter and reduced positive staining regions for MBP were observed in brain sections of an LPS-induced rat model of periventricular leukomalacia (PVL) [15]. Contrary to our expectations, we observed that the expression of MBP in the hippocampus was comparable between the three groups at adolescence (Figure 3a,b). This phenomenon was also confirmed at the mRNA level (Figure 3c).

### 2.5. hAFSC Administration Improved Spatial Awareness and Memory-Based Behavior

**Figure 4:**
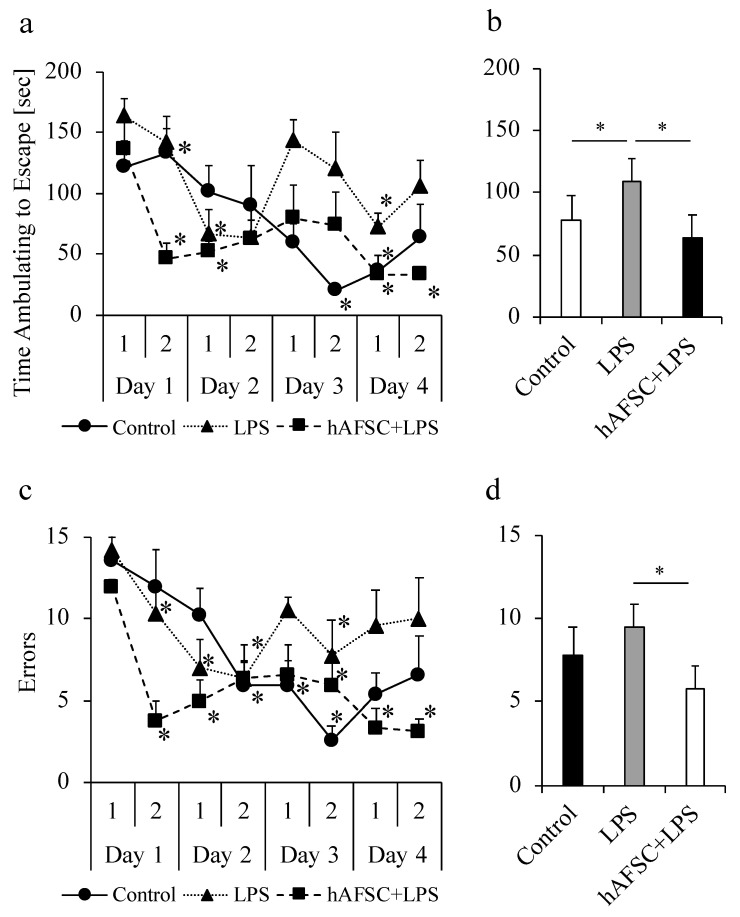
Results of Barnes maze for rats. Rats of all groups were subjected to Barnes maze, 2 weeks after lipopolysaccharide (LPS) administration. Performance of rats in the control group (*n* = 5), LPS group (*n* = 5), and human amniotic stem cells (hAFSC+LPS) group (*n* = 5) in the Barnes maze Trials 1–8. Results are means ± SD (*n* = 5). * *p* < 0.05 compared with the corresponding data in the first trial on day 1 (**a**) and (**c**). Over the course of Trials 1–8, rats in the LPS group took longer to escape (**b**) and made more errors (**d**) than did rats in the control group; the performance was improved in the hAFSC+LPS group. ANOVA with repeated measures showed these differences to be significant in all the cases (* *p* < 0.05).

We analyzed the ability of rats to find a target box to assess their learning, following the method described previously [16,17]. In all the three groups, the time to find the target box and the number of errors were significantly decreased as the number of trials increased for each animal (Figure 4a,c). The animals appeared to explore the platform randomly in the first trial on the first day, and by the fourth day, they were able to gradually refine their search patterns and went directly to escape. These results suggest that the animals showed spatial learning ability. We then compared the mean time taken for exploration for a total of eight trials. The results of this experiment showed that the time required for the search was significantly prolonged in the LPS group compared with that in the control group, and the prolongation was significantly attenuated by hAFSC treatment (Figure 4b, *p* < 0.05 in all the cases). The analysis of the number of errors showed a trend similar to that seen for the time required for ambulating to escape (Figure 4d). The number of errors significantly increased in the LPS group compared to that in the control group, and the increase was significantly attenuated by hAFSC treatment.

## 3. Discussion

Although concerns have been raised that improved survival of infants treated with hAFSCs might be accompanied by an increase in disabling morbidity in neonatal sepsis survivors, in this study, we found that prophylactic therapy with hAFSCs improved cognitive impairment in neonatal sepsis survivors at adolescence in rats. Together with our findings in a previous study [14], herein, we prove that hAFSC treatment improves mortality and morbidity in neonatal sepsis. The treatment suppresses acute reactive gliosis, such as astrocyte and microglial activation, and subsequently reduces astrogliosis in the hippocampal region for a long time, and could result in a favorable long-term neurological outcome.

It is essential to identify whether prophylactic treatment with hAFSCs improves survival without major morbidities for developing new clinical strategies. This would ensure that the therapy not only reduces mortality, but is also effective in improving the quality of life in neonatal sepsis survivors. Because brain damage in neonatal sepsis survivors can be induced by a variety of factors, including preterm birth, inflammation, infection, hypoxia, and ischemia [4], in the present study, we investigated the therapeutic potential of hAFSCs in the developing central nervous system with an emphasis on neuroinflammation. Previous preclinical reports indicate that LPS administered at P3–4 causes acute inflammation involving reactive astrogliosis associated with microstructural alterations in the developing white matter [18]. In this study, we used a neonatal rat model of LPS administration to induce systemic inflammation in the whole body. In particular, the procedure evoked neuroinflammation, as indicated by reactive gliosis [4,19]. Because the hippocampal region plays a central role in regulating spatiotemporal and cognitive functions and is susceptible to injury by ischemia and inflammation [20,21,22], we investigated this region in the present study. We found that hAFSC administration significantly attenuated LPS-induced reactive gliosis in the hippocampal region in the early stages. Thereafter, microgliosis spontaneously resolved in the chronic phase regardless of the hAFSC treatment. On the contrary, astrogliosis lasted for a longer time without hAFSC treatment than it did with hAFSC treatment. These results suggest that hAFSCs suppress neuroinflammation following the suppression of the inflammatory reaction in the whole body in the early phase, resulting in reduced astrogliosis and improved spatial awareness and memory-based behavior at adolescence.

Because the brains of 3-day old neonatal rats are known to be immature and vulnerable [23,24], inflammation of brain during this period persists into the adulthood and is associated with cognitive impairment [25,26,27]. In line with previous reports, we demonstrate that LPS-induced inflammation in 3-day old neonatal rats leads to the impairment of spatial awareness and memory-based behavior at adolescence using the Barnes maze, which is one of the most frequently used methods to investigate cognitive functions [28,29,30,31]. These findings can provide a basis for the higher rate of cognitive and memory impairment in longitudinal studies of human sepsis survivors [32]. In this study, we found that prophylactic therapy with hAFSCs improved cognitive impairment in neonatal sepsis survivors at adolescence in rats, followed by the resolution of histological neuroinflammation in the hippocampus. Together with our previous findings, we confirm that hAFSCs have the therapeutic potential to improve mortality and morbidity by ameliorating inflammation in neonatal sepsis.

PVL, characterized by impaired myelination, results in the maldevelopment of oligodendrocytes, leading to cerebral palsy [33]. However, we could not show both impaired myelination after LPS challenge and the therapeutic effect of hAFSCs on the expression of MBP in the rat brain. Previously, MSCs or MSC-derived extracellular vesicles were shown to have the potential to ameliorate PVL in rats at postnatal day 11 (P11) or P12. In these reports, the authors investigated the therapeutic effect of MSCs or MSC-derived extracellular vesicles on MBP expression using a PVL model created by exposure of 3- or 4-day-old rats to LPS, similar to our neonatal sepsis model. In contrast to these reports, in our study, MBP expression in the hippocampus was comparable between hAFSCs+LPS and LPS groups at adolescence. There are several factors that might have contributed to these differences between the outcomes of the present and previous studies. First, in our study, the reduction in the expression of MBP in neonatal sepsis survivors might have already been compensated for by endogenous mechanisms. Another possibility is the difference in the areas that were observed. We determined the expression of MBP in the hippocampus, whereas in previous studies, the expression was determined in the corona radiata and corpus callosum. Finally, the differences could be due to the difference in the number of doses. We administered a single dose of hAFSCs, whereas Dormmelschmidt et al. reported the results of administering two doses of MSC-derived extracellular vesicles. Repeated administration of hAFSCs might further reduce reactive astrogliosis, restore myelination deficits, and improve neurological outcomes.

This study has several limitations. First, the Barnes maze is quite lengthy to perform behavioral analysis on newborns. As we could not conduct an assessment of behavior and memory functions during the neonatal period, we could not investigate the changes in the same individuals from the neonatal to the adolescent stage. Next, it is impossible to explain the therapeutic effect of hAFSCs by reduction of reactive gliosis in the hippocampus alone. Further studies are required to clarify the detailed mechanisms underlying the protective potential of hAFSCs.

To the best of our knowledge, this is the first report to describe the concept that treatment with hAFSCs improves cognitive impairment in neonatal sepsis survivors. Together with our previous findings that prophylactic therapy with hAFSCs improves survival in neonatal sepsis through immunomodulation, the results of the present study show that hAFSCs have the therapeutic potential to reduce the mortality and morbidity associated with neonatal sepsis.

## 4. Materials and Methods

### 4.1. Isolation, Culture, and Immunophenotypic Characterization of CD117+ Amniotic Fluid Cells

hAFSCs were isolated from amniotic fluid using our previously reported method [14]. Briefly, the collected amniotic fluid was centrifuged and the amniotic fluid cells were isolated. After the cells were cultured, CD117-positive cells were isolated using a magnetic cell sorting kit (Miltenyi Biotec, Auburn, CA, USA). According to the criteria defined by the International Society for Cellular Therapy for MSCs, the obtained cells were confirmed to be positive for mesenchymal markers and negative for hematopoietic markers by flow cytometry and to have the potential to differentiate into adipocytes, osteocytes, and chondrocytes. Subsequently, hAFSCs were cultured in the growth medium, which was composed of α-modified Eagle minimum essential medium (αMEM; Invitrogen, Carlsbad, CA, USA), 15% fetal bovine serum (FBS) (Invitrogen, Carlsbad, CA, USA), 1% L-glutamine (Invitrogen, Carlsbad, CA, USA), 1% penicillin/ It consisting of streptomycin (Invitrogen, Carlsbad, CA, USA), and 40% AmnioMax-II (Life Technologies, Carlsbad, CA, USA), and used in the experiments.

### 4.2. Animals

All experiments were approved by the Animal Committee of Keio University, Japan (no. 18003-(0) and -(1)). We created a rat model of LPS-induced neonatal sepsis as described previously [14,34]. Briefly, male Sprague–Dawley (SD) rat pups (Charles River Laboratories Japan Inc., Kanagawa, Japan) at P3 were administered an intraperitoneal (i.p.) injection of 0.25 mg/kg LPS (*Escherichia coli* O55: B5, Sigma-Aldrich, Steinheim, Germany) dissolved in saline. A total of 40 animals were prepared, of which 15 animals that survived over 48 h after LPS administration were used in subsequent experiments (survival rate at 48h after LPS administration without hAFSCs pretreatment: 37.5% (15/40)). As a prophylactic treatment, hAFSCs dissolved in saline were administered i.p. 3 h before LPS administration (survival rate at 48 h after LPS administration with hAFSCs pretreatment: 53.5% (15/28)). The sham intervention was performed using the same amount of saline i.p. (survival rate at 48 h after the sham intervention: 100% (15/15)).

At 48 h and 4 weeks after LPS administration, the rats were sacrificed and their brains were removed. The removed specimens were fixed by immersing in 4% paraformaldehyde (PFA) for 24 h. Subsequently, a cryostat was used to make 7 µm sections of frozen samples that included the hippocampus. We also conducted a 4-day behavioral study using the methods described in the next section, starting 4 weeks after LPS treatment. Subsequently, sections of the brain were prepared in the same manner as described above, 6 weeks after LPS administration.

### 4.3. Immunohistochemical Analysis

Astrocytes and microglial cells were assessed using anti-GFAP antibodies (Dako Corporation, Carpinteria, CA, USA) and Iba-1 (Wako, Osaka, Japan, 1:100). Nuclei were counterstained with Hoechst 33342 (Wako, Osaka, Japan, 1:100).

Images were captured using a BZX-810 camera (Keyence, Osaka, Japan), and morphometric analysis was performed using the ImageJ software (https://imagej.nih.gov/ij/). To assess the extent of neuroinflammation, we counted GFAP-and Iba-1-positive areas in the brain sections.

### 4.4. RNA Extraction and Real-Time RT-PCR Analysis for MBP

For total RNA extraction, periventricular white matter tissue was removed from the total coronal sections, approximately −1 to +1 mm from the bregma. Tissues were immediately immersed in RNAlater solution (Invitrogen, Carlsbad, CA, USA) and stored at 4 °C until they were used for experiments. The tissues were homogenized using QIAshredder spin columns (Qiagen Inc., Hilden, Germany). Total RNA was isolated using the RNeasy mini kit (Qiagen Inc., Hilden, Germany) according to the manufacturer’s instructions. Reverse transcription of total RNA was performed using the Prime Script RT Master Mix (Takara Bio Inc., Shiga, Japan). Primers used in real-time PCR were as follows: *MBP*: 5′-CTC TGG CAA GGA CTC ACA CAC-3′ (forward) and 5′-TCT GCT GAG GGA CAG GCC TCT C-3′ (reverse). Quantitative PCR was performed using SYBR Premix Ex Taq II (Tli RNaseH Plus; Takara Bio, Shiga, Japan) on the Bio-Rad CFX96 Real-Time PCR System (Bio-Rad, Richmond, CA, USA). The assay was performed in duplicate for each sample. The relative gene expression in each sample was analyzed using the 2^−ΔΔCT^ method. The gene expression values were normalized to those of *β-actin*, which was used as an internal control.

### 4.5. Barnes Maze Testing

We used an apparatus based on the one first described by Barnes [30]. The Barnes maze is designed to test spatial learning and memory in rodents. The top panel of the maze was made of circular white acrylic (5 mm thick) with a diameter of 1220 mm, and had 20 evenly spaced holes, 10 cm in diameter, at the edges (Sugahara Kogei Co., Ltd., Chiba, Japan, Figure S1A). The top panel was placed on a desk at a height of approximately 150 cm above the ground. This is the height from which an animal cannot voluntarily jump down to the ground. At the end of the platform, under one of the holes, there was a dark box where the rat could hide. This box, called the target box, was kept in a constant position relative to the room throughout the experiment. Two 32-watt fluorescent lights (National FHF32EX-N-H; Panasonic Co., Osaka, Japan) with reflectors were installed directly above the maze [31]. Various objects (large metal door, lab bench with microscope, sink, rearing cage rack, and an in vivo imaging system (IVIS, Caliper Life Sciences, Hopkinton, MA, USA) were set up around the maze, which provided ample spatial cues for the rats (Figure S1B,C). Based on these spatial cues, the rats were encouraged to enter the target box as quickly as possible to escape the bright light.

The experimental method was based on that described in previous studies [16,17]. Animals were made to perform two trials, with a 15-min gap between trials; the trials were performed on four different days. In each trial, the animal was allowed to stay in the maze for a maximum of 3 min and find the target box. The errors (indicated by the animal placing its nose or paws on the edge of a hole that was not connected to the target box) and the time taken to reach the target box were measured for each trial. Once the animal reached the target box, it was allowed to remain there for 2 min and then moved to its rearing cage and allowed to rest for 15 min; it was subsequently returned to the maze for another trial. Animals that failed to find the target box within 3 min were manually guided to the box by the experimenter and were allowed to stay there for 2 min. After each test, the Barnes maze was cleaned with paper towels wet with 70% ethanol to eliminate odor cues.

### 4.6. Statistical Analysis

All values are expressed as means ± standard error. Statistical differences between groups were assessed using analysis of variance and Tukey’s honest significant difference. All analyses were performed with the SPSS Statistics software (Version 25, IBM Inc., Armonk, NY, USA). *p* values less than 0.05 were considered statistically significant.

## 5. Conclusions

Severe life-threatening neonatal sepsis worsens neurological prognosis. However, the anti-inflammatory effects of hAFSCs could reduce neuroinflammation in the brain and improve spatial awareness and memory-based behavior. Together with our previous findings, the results of the present study show that hAFSCs have therapeutic potential to reduce the mortality and morbidity associated with neonatal sepsis. The novel treatment strategy using hAFSCs could be effective for neonatal sepsis.

## Supplementary Materials

Supplementary materials can be found at https://www.mdpi.com/1422-0067/21/24/9590/s1.

## Abbreviations

DG	Dentate gyrus
GFAP	Glial fibrillary acidic protein
MSCs	Mesenchymal stem cells
PVL	Periventricular leukomalacia
hAFSCs	Human amniotic fluid stem cells
Iba-1	Ionized calcium-binding adapter molecule 1
