# Supplementary material for: Prophylactic Therapy with Human Amniotic Fluid Stem Cells Improves Long-Term Cognitive Impairment in Rat Neonatal Sepsis Survivors
Source: Int J Mol Sci. 2020 Dec 16;21(24):9590. doi: 10.3390/ijms21249590 (PMC7766081; doi:10.3390/ijms21249590)
Supplement: Supplementary file 1 [file ijms-21-09590-s001.pdf]

Fig. S1

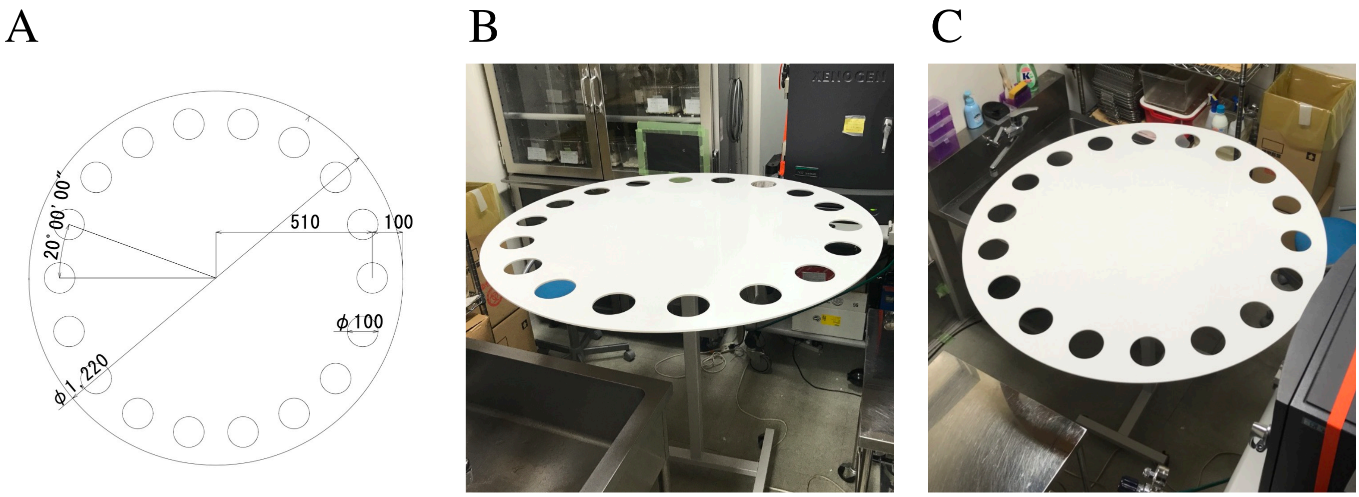

Fig. S1. Blueprint used to cut out from an acrylic sheet (A). A view of the Barnes maze set up at the site of the experiment. There are some spatial recognition clues for rats around the Barnes maze (B, C).
